# Potent restriction of HIV-1 and SIV_mac239_ Replication by African Green Monkey TRIM5α

**DOI:** 10.1186/s12977-015-0137-9

**Published:** 2015-02-07

**Authors:** Lori V Coren, Matthew T Trivett, Sumiti Jain, Victor I Ayala, Gregory Q Del Prete, Claes Ohlen, David E Ott

**Affiliations:** AIDS and Cancer Virus Program, Leidos Biomedical Research Inc., Frederick National Laboratory for Cancer Research, PO Box B, Frederick, MD 21702 USA

**Keywords:** HIV, SIV, TRIM5α, Restriction factor, Cumulative restriction

## Abstract

**Background:**

The TRIM5α protein is a principal restriction factor that contributes to an HIV-1 replication block in rhesus macaque CD4^+^ T cells by preventing reverse transcription. HIV-1 restriction is induced in human CD4^+^ T cells by expression of rhesus TRIM5α as well as those of other old world monkeys. While TRIM5α restriction has been extensively studied in single-round infection assays, fewer studies have examined restriction after extended viral replication.

**Results:**

To examine TRIM5α restriction of replication, we studied the ability of TRIM5α proteins from African green monkey (AgmTRIM5α) and gorilla (gorTRIM5α) to restrict HIV-1 and SIV_mac239_ replication. These xenogeneic *TRIM5α* genes were transduced into human Jurkat-CCR5 cells (JR5), which were then exposed to HIV-1 or SIV_mac239_. In our single-round infection assays, AgmTRIM5α showed a relatively modest 4- to 10-fold restriction of HIV-1 and SIV_mac239,_ while gorTRIM5α produced a 2- and 3-fold restriction of HIV-1 and SIV_mac239_, respectively, consistent with the majority of previously published single-round studies. To assess the impact of these modest effects on infection, we tested restriction in replication systems initiated with either cell-free or cell-to-cell challenges. AgmTRIM5α powerfully restricted both HIV-1 and SIV_mac239_ replication 14 days after cell-free infection, with a ≥ 3-log effect. Moreover, expression of AgmTRIM5α restricted HIV-1 and SIV_mac239_ replication by 2-logs when co-cultured with infected JR5 cells for 12 days. In contrast, neither expression of gorTRIM5α nor rhesus TRIM5α induced significant resistance when co-cultured with infected cells. Follow up experiments showed that the observed differences between replication and infection were not due to assembly defects as xenogeneic TRIM5α expression had no effect on either virion production or specific infectivity.

**Conclusions:**

Our results indicate that AgmTRIM5α has a much greater effect on extended replication than on any single infection event, suggesting that AgmTRIM5α restriction acts cumulatively, building up over many rounds of replication. Furthermore, AgmTRIM5α was able to potently restrict both HIV-1 and SIV replication in a cell-to-cell infection challenge. Thus, AgmTRIM5α is unique among the TRIM5α species tested to date, being able to restrict even at the high multiplicities of infection presented by mixed culture with nonrestrictive infected cells.

## Background

Studying cellular resistance to HIV-1 infection mediated by cellular proteins, i.e. resistance factors, is important for the understanding of viral biology and identifying potential opportunities for AIDS therapeutics [1-10]. These cellular proteins interact with viral partners to block various steps in the retroviral replication cycle, thereby suppressing virus infection and spread [11]. The TRIM5α restriction factor belongs to the very large tripartite motif (TRIM) family of proteins [12,13], and is a well-studied HIV-1 resistance factor [14]. In the early 1990’s, a resistance to HIV-1 infection of rhesus macaque cells was observed, manifested as a post-entry block to reverse transcription that mapped to a few viral proteins, most notably Gag [15-17]. Over a decade later, the TRIM5α cytoplasmic body protein, was identified as a principal restriction factor for HIV-1 in rhesus macaque CD4^+^ T cells which binds the capsid protein (CA) in capsid cores after virus entry, thereby, interfering with early reverse transcription [18,19]. In many cases viral restriction in normally permissive cells can be produced by ectopic expression of TRIM5α from other species, i.e., xenogeneic expression, producing a somewhat complicated pattern of restriction in a variety of virus/TRIM5α pairings [18,20-26].

As with all TRIM family members [12,13], TRIM5α contains a really interesting new gene (RING) finger domain with an E3 ubiquitin ligase activity, a B-box zinc finger, a coiled-coil domain as well as a fourth non-canonical B30.2(SPRY) domain [12,13]. The basis for the differential xenogeneic restricting capabilities of TRIM5α proteins lies in the RING and B30.2(SPRY) domains. The B30.2(SPRY) region binds capsid and is necessary, but not sufficient for restriction [9,18,27-30]. A single amino acid difference in the RING finger is critical for the restriction of SIV_mac_ by *tantalus* African green monkey TRIM5α (AgmTRIM5α) [31,32], but not other TRIM5α/virus combinations [18,29,33,34]. Thus, the contributions of the RING domain across the different TRIM5α/virus combinations are quite complicated and, in some cases, unclear. Also, the exact nature of the block is clouded by data supporting the possibility of multiple mechanisms of interference with the post-entry infection process that act between early reverse transcription [18] and nuclear entry/integration of the cDNA [35,36].

TRIM5α restricts infection inside the cell by binding the CA-coated capsid core structure soon after entry [37]. The capsid core contains all of the elements needed for infection, the genomic RNA bound by nucleocapsid protein, reverse transcriptase, and integrase, all encased in a highly structured conical CA protein shell poised to carry out the infection process [38]. Current models propose infection proceeding, post-entry, by the CA core rearranging and partially uncoating in a controlled manner at the appropriate time to allow for reverse transcription. Therefore, CA-CA interactions in the capsid core need to be finely balanced, strong enough to maintain core structure *in virio*, yet weak enough to appropriately uncoat during the infection process inside the cell. Indeed, virion infectivity requires the correct conical capsid core structure [39]: CA mutations that either stabilize or destabilize the capsid core have drastic effects on infectivity, reflecting the fragile balance of core stability [40].

The exact mechanism for TRIM5α restriction is not completely understood, but it clearly involves TRIM5α binding the CA shell in a highly cooperative manner, which results in the proteins forming an ordered cage around the core [41,42]. The current data supports two models for the mechanism of TRIM5α restriction. In one model, the TRIM5α cage disrupts the normal, presumably ordered, rearrangement and uncoating process, thereby prematurely disintegrating the CA shell [35,43-45]. Alternatively, the RING finger E3 ubiquitin ligase activity of TRIM5α could be ubiquitinated itself as well as CA after complex formation, marking both TRIM5α and the CA core for destruction by the proteasome [32,36]. Indeed, TRIM5α and viral components are associated with cytoplasmic bodies that accumulate upon proteasome inhibition [46], although CA ubiquitination by TRIM5α has not yet been observed. It is important to note that these two models are not mutually exclusive and there is strong evidence that different TRIM5α/virus combinations could use different mechanisms (please see references in [32,36,42]). For instance, HIV-1 restriction by rhesus macaque TRIM5α (rhTRIM5α) does not require the RING E3 ubiquitin ligase activity [35], while this function is required for restriction of SIV_mac_ by AgmTRIM5α [32]. Finally, TRIM5α acts as a pattern recognition receptor that senses TRIM5α-capsid core complexes, which then activates innate immune signaling [47]. While its exact contribution to restriction is not clear, this induction of signaling is required for TRIM5α restriction in some experimental contexts [47].

Normally, human TRIM5α restricts only a few retroviruses, being ineffective against HIV-1, SIV_mac_ and SIV_Agm_ [22,24,25], although transduction of a mutant human TRIM5α with an extended half-life can induce HIV-1 restriction in human cells [10]. In contrast, single-round infectivity assays with pseudotyped defective viruses or viral vectors have found that rhTRIM5α strongly (>70- 95%) restricts HIV-1 infection, while AgmTRIM5α weakly (~60%) restricts SIV_mac_, with gorilla TRIM5α (gorTRIM5α) having a stronger effect (~90%) [22]. Despite a large body of infection experiments, only a few studies have examined TRIM5α restriction using wild-type viruses in cell-free replication assays [8,10,18,30,48,49].

TRIM5α restriction provides a potent block to HIV-1 infection of rhesus macaque CD4^+^ T cells that, along with other resistance factors, make them essentially unable to support a spreading infection [17,23,48,50]. Because of this effect, xenogeneic expression of rhTRIM5α by gene transfer has been proposed as a way to protect human CD4^+^ T cells from HIV-1 [1,3-5]. Despite its promise, preliminary work indicated that xenogeneic expression of rhTRIM5α protected primary human CD4^+^ T cells only under certain circumstances [48], most notably when all of the cells expressed the restricting protein. rhTRIM5α failed to protect cells from HIV-1 exposure by cell-to-cell contact with infected cells without rhTRIM5α [48]. To better investigate the basis for TRIM5α restriction of replication and extend TRIM5α-mediated restriction studies to the SIV/rhesus macaque model system, we chose to examine two lesser-studied TRIM5α proteins that were previously found to restrict SIV_mac_, AgmTRIM5α and gorTRIM5α, for their ability to restrict either HIV-1 or SIV_mac239_ replication in a human CD4^+^ T-cell line. Our results showed that, despite a relatively weak effect against HIV-1 and SIV_mac_ viruses and pseudotyped vectors in short term and single-round infection assays, AgmTRIM5α expression induced a high level of restriction to both HIV-1 and SIV replication in a human CD4^+^ T-cell line in both cell-free and cell-to-cell challenge systems.

## Results

**Figure 1:**
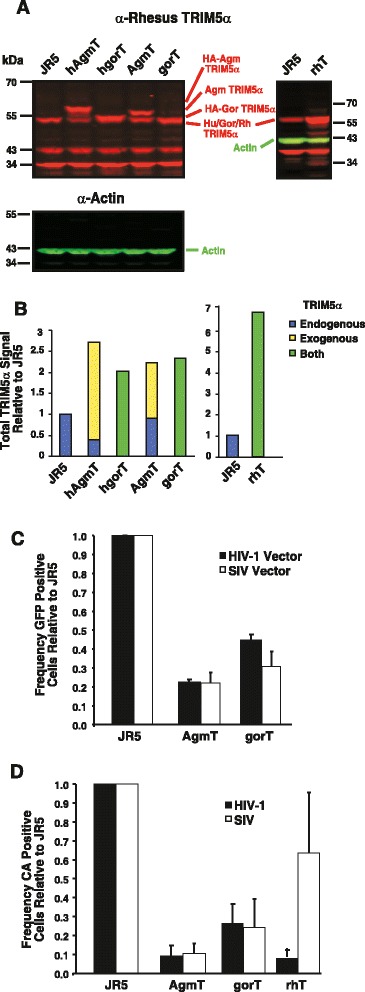
**Ecotopic expression in JR5 cells and infection assays. (A)**. A NIr immunoblot of cell lysate samples is presented with TRIM5α signal in red and actin in green. Samples are identified above their respective lanes, with molecular mass standard sizes and band identities displayed at the margins. **(B)**. A graph of fluorescent immunoblot signal intensities in the NIr blot relative to those of the untransduced JR5 cells is presented with the amount of endogenous, human TRIM5α-colored blue, xenogeneic TRIM5α colored yellow, and co-migrating TRIM5α proteins colored green. **(C)** A graph of flow cytometry analysis results for GFP signal from single-round infectivity assays (MOI = 0.05) using HIV-1_NL4-3_ and SIV_mac239_-expressing GFP vectors relative to the values for the JR5 positive controls is presented. **(D)** A graph of results of flow cytometry analysis for intracellular CA 40-h post infection with either HIV-1_NL4-3_ or SIV_mac239_ (MOI = 0.05) is presented. Graphs C and D are averages of three independent experiments and error bars indicate standard deviation. Statistical of the experimental versus the control values for the data in both panels **C** and **D** generated *p*-values of <0.012 for all pairs except for the rhTRIM5α/SIV sample, *p* = 0.37.

To study TRIM5α restriction, we produced murine retroviral vectors that express N-terminally hemagglutinin epitope (HA) tagged TRIM5α proteins from either *tantalus* African green monkey (SMS-hAgmT) or gorilla (SMS-hgorT) [21,22] along with the GFP and the puromycin resistance genes. Because, N-terminal HA tags might affect the function of TRIM5α [20], we also produced two vectors (Babe-AgmT and Babe-gorT) that express native TRIM5α proteins and the puromycin-resistance gene. JR5 cells (human Jurkat CD4^+^ T cells transduced with the *CCR5* gene) were transduced with pseudotyped vectors and puromycin resistant cells were selected, producing the hAgmT, hgorT, AgmT, and gorT cell lines. To measure the expression of ecotopic TRIM5α in these JR5 cell lines, we analyzed cell lysates by immunoblotting using the quantitative two-color near infrared fluorescence (NIr) LI-COR system with the 3F1-1-9 monoclonal antibody specific for a primate-conserved rhTRIM5α epitope and an actin antibody as a cell lysate loading control. The results (Figure 1A) showed that, in addition to the endogenous human TRIM5α band at 56 kDa (present in the untransduced JR5 cell lysate), there were bands at 59 and 57 kDa in the hAgmT and hgorT lysates, respectively, corresponding to the expected molecular masses (TRIM5α with the HA-tag) of the hAgmTRIM5α and hgorTRIM5α proteins. Similarly, the AgmT cell lysates contained bands at 56 kDa and 58 kDa, consistent with human and AgmTRIM5α proteins, respectively. In contrast, the gorT line contained only one band at 56 kDa, yet with a greater intensity relative to the bands in the other samples (Figure 1A). Due to their nearly identical molecular mass, ectopic gorilla and endogenous human TRIM5α proteins co-migrate. Measurement of the fluorescence intensities of both the xenogeneic and endogenous TRIM5α bands and normalization by actin band signal revealed that the range of ectopic TRIM5α expression was close to normal physiological levels (Figure 1B), only 1- to 2-fold over that of endogenous human TRIM5α among the different transduced cell lines.

### Xenogeneic TRIM5α expression modestly restricts HIV-1 and SIV single-round infection

The magnitude of TRIM5α restriction has been well established by challenging cells with viral vectors or defective GFP-expressing viruses [18,20-26]. To confirm the viral inhibition effect of xenogeneic TRIM5α on single-round infection in our cell lines, the AgmT and gorT cells were exposed to either HIV-1 or SIV-based lentiviral vectors that express GFP. Flow cytometry for GFP fluorescence revealed that AgmT cells exhibited somewhat more resistance to HIV vector transduction than the gorT cells, with a 4-fold compared to a 3-fold resistance to infection, respectively (Figure 1C). These results generally agree with prior published studies. To examine infection in a more physiological system, the xenogeneic TRIM5α-expressing cells were infected with either HIV-1 or SIV_mac239_ and Gag-positive cells were measured 40-h post-infection by intracellular flow cytometry with CA antibodies. To control for non-specific virion binding, the numbers of Gag-positive cells were adjusted for the background present in the heat-inactivated virus. The results were similar to those of the vector-derived data: the infection of AgmT cells was 90% lower for both viruses, and gorT cells demonstrated a more modest reduction of infection, approximately 70% (Figure 1D).

For comparison to the more extensively studied rhesus macaque system, we examined the effect of xenogeneic rhTRIM5α in our short-term infection assay. JR5 were transduced with the Babe-rhT vector, which expresses rhTRIM5α and puromycin acetyltransferase, and selected with puromycin to produce the rhT cell line. Immunoblot analysis of rhT lysates revealed only a single, more intense TRIM5α-sized band due to the close molecular masses of rhTRIM5α and its human counterpart (Figure 1A). Measurement of the bands found a 6-fold increase in the TRIM5α over that of the JR5 controls (Figure 1B), a higher level of exogenous expression that the other JR5/TRIM5α cell lines. Consistent with prior results, the rhTRIM5α-expressing cells reduced HIV-1 infectivity by an amount similar to that of the AgmTRIM5α proteins in the short term assay and did not significantly affect SIV infection, as expected (Figure 1D). Modest restriction was also observed by assaying HIV-1 and SIV_mac239_ in Agm- and gorTRIM5α-expressing TZMbl cells (data not shown). Thus, in our systems, xenogeneic expression of these three TRIM5α proteins results in a modest level of restriction in single-round/short-term assays and recapitulates most prior studies of Agm- and rhTRIM5α [9,18,21-24,26-30].

### Strong AgmTRIM5α replication restriction after a cell-free challenge

**Figure 2:**
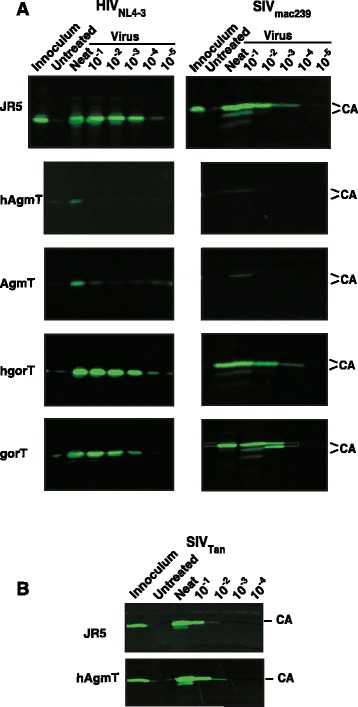
**Cell** **-** **free infection challenge assays.** Representative CA NIr immunoblots of HIV-1 and SIV virions in infected cell culture supernatant samples from three independent experiments are presented. Virus and infection dilutions are labeled above the blot. Samples of the viral inocula (first lane), and supernatant samples from untreated cells (second lane) and from 14-day cultures infected at different dilutions (lanes 3–8) are presented above the blot panels. **(A)**. Results from HIV_NL43_ (left) and SIV_mac 239_ (right), and **(B)** from SIV_AgmTan-1_.

To determine the effect of these TRIM5α proteins on extended replication, JR5, hAgmT, AgmT, hgorT, and gorT cells were infected with cell-free stocks of HIV-1_NL4-3_ or SIV_mac239_ from freshly prepared transfection supernatants at 10-fold dilutions. Cells were cultured for 14 days with periodic fluid changes before supernatants were harvested and analyzed for the presence of virions by CA NIr immunoblot (Figure 2A). The HIV-1 cell-free challenge assays showed that the JR5 cells had detectable p24^CA^ bands out to the 10^-4^ inoculum dilution, with no detectable signal in the 10^-5^ dilution, indicating an end-point titer of ≥ 10^4^ to < 10^5^. In contrast, supernatants from the hAgmT and AgmT cultures had a slight p24^CA^ signal in the neat infection sample and no detectable signal in any of those from virus dilution cultures (Figure 2A), indicating a strong level of restriction induced by both versions of TRIM5α, ≥ 10^3^/<10^4^ fold. In contrast, the hgorT and gorT cultures had essentially the same levels of p24^CA^ in the challenge dilutions as the JR5 positive controls, with intense p24^CA^ bands in the neat through the 10^4^ dilution samples, indicating at least levels of replication similar to those of JR5 (Figure 2A). This is expected given the high level of conservation of between human and gorilla TRIM5α genes [22].

A NIr immunoblot of virus samples from JR5 cultures infected with the SIV_mac239_ dilution series revealed that p28^CA^ signals were detectable out to the 10^-3^ dilution (Figure 2A), in part reflecting a lower susceptibility of JR5 cells to infection by SIV_mac239_ than to HIV-1, a phenomenon that we observe in most human cell lines (D. Ott unpublished data). Similar to the HIV-1 results, both hAgmT and AgmT cells showed a high level of resistance with no p28^CA^ signal detectable in any of the supernatants from infected cells (Figure 2A), indicating a resistance of ≥10^3^. The cell-free SIV-challenged gorT cells had little p28^CA^ signal at the 10^-3^ dilution compared to that of the JR5 control band while the hgorT had little difference in intensity from the control at the 10^-3^ dilution (Figure 2A), indicating, at most, a 10-fold level of restriction induced by gorTRIM5α, considerably lower than that of AgmTRIM5α transduced cells.

Others have noted that overexpression of TRIM5α to high levels (>50-fold) [20,51,52] or increasing the stability of TRIM5α [10] can result in restriction-like effects in single-round experiments. While the amount of total TRIM5α expression, both endogenous and exogneous, in the hAgmT and AgmT cells was elevated only 2-to- 2.5-fold in the transduced cell lines, it is possible that these levels of over-expression might still cause nonspecific restriction. To address this possibility, we examined both JR5 and hAgmT cells in our replication assay with SIV_AgmTan-1_, a cognate virus for AgmTRIM5α. The results showed no evidence of restriction in the hAgmT cells, with nearly similar amounts p28^CA^ signal detected in the neat and 10^-1^ dilution samples and a slight signal detected in the 10^-2^ dilution sample (Figure 2B). Thus, the restriction patterns observed in these experiments are not an artifact of TRIM5α overexpression and are consistent with specific AgmTRIM5α-mediated restriction of HIV-1 and SIV_mac239_. Taken together, these results indicate that AgmTRIM5α specifically restricts HIV-1 and SIV, while gorTRIM5α fails to restrict HIV-1 and only weakly restricts SIV.

### AgmTRIM5α restricts when challenged with infected cells

**Figure 3:**
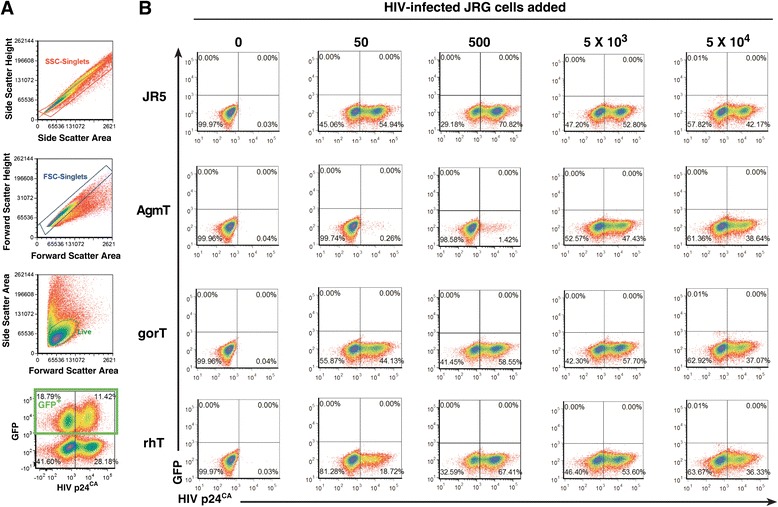
**Flow cytometry analysis of HIV**_**NL4-3**_ **cell** **-** **to** **-** **cell challenge experiments.** Representative analyses of 12-day mixed-cell cultures from three independent experiments are presented. **(A)**. Gating of mixed cell cultures (from top to bottom) for single cells, side scatter height by area and forward scatter height by area plots, and for live cells, side scatter area by forward scatter area plots. An example of the gating of the input infected JR5G cells from the JR5 and TRIM5α cultures is presented in the bottom plot (the 5 × 10^5^ cells HIV-JR5G and 5 × 10^5^ JR5 mixed culture shown). **(B)**. Flow cytometry analysis gating on the GFP^–^ cells in the mixed cultures are presented with HIV CA staining on the X-axis and GFP fluorescence in the Y-axis. Each series of 5 × 10^5^ TRIM5α cultures are identified at left and the infected JR5G cells added are indicated over each challenge group.

**Figure 4:**
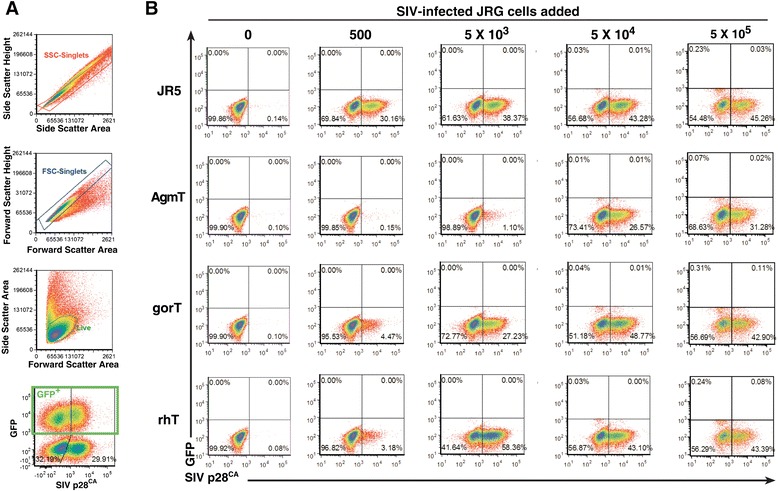
**Flow cytometry analysis of SIV**_**mac239**_ **cell** **-** **to** **-** **cell challenge experiments.** Representative analyses of 12-day mixed-cell cultures from three independent experiments are presented. **(A)**. Gating of mixed cell cultures (from top to bottom) for single cells, side scatter height by area and forward scatter height by area plots, and for live cells, side scatter area by forward scatter area plots. An example of the gating of the input infected JR5G cells from the JR5 and TRIM5α cultures is presented in the bottom plot (the 5 × 10^5^ cells SIV-JR5G and 5 × 10^5^ JR5 mixed culture shown). **(B)**. Flow cytometry analysis gating on the GFP^–^ cells in the mixed cultures are presented with SIV CA staining on the X-axis and GFP fluorescence in the Y-axis. Each series of 5 × 10^5^ TRIM5α cultures are identified at left and the infected JR5G cells added are indicated over each challenge group.

The cell-free virus challenge results presented above demonstrate restriction in a culture in which all of the cells express xenogeneic TRIM5α. However, cell-to-cell infection, i.e., extracellular virions being transferred across cell–cell contacts, is up to 1000-fold more efficient than a cell-free challenge, [48,53-61]. To test restriction in our xenogeneic TRIM5α-expressing cells by this more stringent challenge, AgmT and gorT cells were cultured with either HIV-1 or SIV-infected JR5G cells, JR5 cells that express GFP to differentiate the infected challenge cells from the TRIM5α-transduced target cells (Figures 3A and 4A), at increasing 10-fold ratios, ranging from 50- to- 5 × 10^5^ cells. After 12-days, the xenogeneic TRIM5α-expressing cells in the mixed cultures were analyzed for infection by intracellular Gag flow cytometry using CA antibodies and gating on GFP negative cells (Figure 3A).

The intracellular p24^CA^ staining for Gag in the JR5 targets revealed the presence of 30% to 70% HIV-infected JR5 cells in all of the mixed cultures (Figure 3B). Interestingly, the cultures initiated with 500 JR5G-infected cells contained the highest level of infected JR5 targets, 70%, while the 5 × 10^5^ cell culture contained only 30% (data not shown), apparently due to cytopathology induced by the presence of HIV-1 over the 12-day testing period. In contrast to the JR5 infection results, the AgmT target cells had either background or considerably reduced levels of Gag staining at the 50- and 500-infected-cell challenge levels, 0.3% and 1% respectively (Figure 3B). The levels of HIV-infected gorT in the various mixed culture ratios mirrored the JR5 cultures, with only a minor reduction of Gag-positive cells in the 50-cell culture (44%), compared to the Gag-positive cells in the JR5 culture (55%). Thus, gorTRIM5α-expressing cells demonstrated little, if any, cell-to-cell restriction, similar to the cell-free challenge results. In contrast, cells expressing AgmTRIM5α exhibited a 10^2^ restrictive phenotype for HIV-1 replication.

Prior results reported by Richardson et al. expressing rhTRIM5α in primary human CD4^+^ T cells observed that restriction protected transduced cells from HIV-1, but failed to do so when untransduced cells were also present at the initial cell-free inoculation. Additional experiments confirmed that this was due to cell-to-cell transfer of HIV-1 from the infected xenogeneic TRIM5α negative cells to the xenogeneic TRIM5α positive cells [48]. To determine whether rhTRIM5α could restrict HIV-1 in our assay, which is similar to the Richardson assay, we tested rhT cells in our cell-to-cell challenge assay. In contrast to the single-round assay, rhT cells showed only marginal HIV-1 restriction at the 50-cell challenge, 19% infected cells, compared to the JR5 control with 55% infected cells present, and essentially the same frequency of infected cells in the 500-cell cultures (Figure 3B). Thus, similar to the results reported by Richardson et al., our results showed that rhTRIM5α fails to restrict HIV-1 infection in this high multiplicity of infection (MOI) cell-to-cell setting.

Flow cytometry of the SIV-mixed cultures found that, as in our cell-free experiments, the extent of the SIV_mac239_ infection of the target JR5 cells, while robust, was less than the extent of HIV-1 infection: SIV infection was clearly evident at the 500-cell challenge, with 30% of the cells infected (Figure 4B). The AgmT results were quite similar to those of the HIV-1 assay: the 500-cell challenge had background staining and the 5 × 10^3^-cell challenge culture contained considerably reduced levels of infected cells, 1% frequency, versus 38% for the JR5 control (Figure 4B). Some restriction was even evident at the 5 × 10^4^-cell challenge with 27% of the AgmT cells containing Gag compared to 43% of the JR5 cells. As seen in the cell-free infection experiments, the gorT cells displayed lower levels of SIV_mac239_ restriction than AgmT cells, reducing the number of infected cells in the 500-cell mixed culture to 5%, as compared to a 30% frequency for the JR5 control and a 27% to 38% presence of infected cells in the 5 × 10^3^-cell challenge culture. As expected, rhT cells exhibited only slightly fewer infected cells than the JR5 controls (Figure 4). Taken together, these data show that AgmTRIM5α potently restricts both HIV-1 and SIV_mac239_, even in this rigorous cell-to-cell challenge, while, as predicted by the cell-free assays, gorTRIM5α only modestly restricts SIV_MAC239._

### AgmTRIM5α replication restriction effect is not due to lower virus production

**Figure 5:**
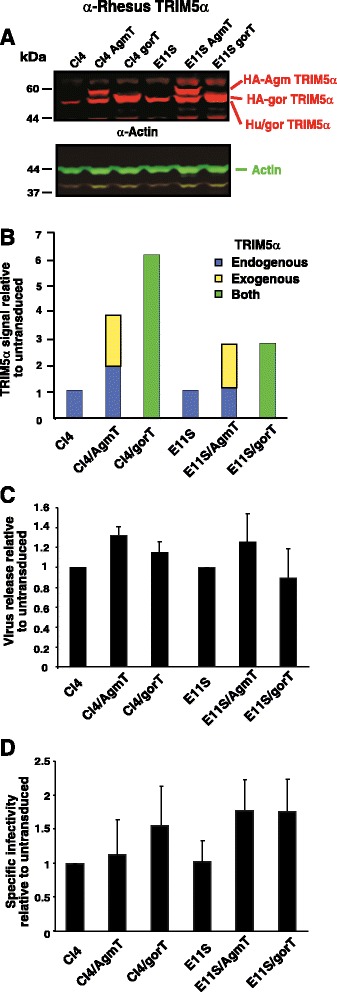
**Ectopic expression of TRIM5α in Clone 4 and E11S cells**, **virion production**, **and infection assays. (A)**. NIr immunoblot of cell lysate samples, with TRIM5α signal in red and actin in green are presented. Samples are identified above their respective lanes. Molecular mass standard sizes are displayed at left and band identities at right. **(B)**. Graph of fluorescent immunoblot signal intensities in the NIr blot relative to those in the untransduced Clone 4 or E11S cells are presented with the amount of endogenous, human TRIM5α-colored blue, xenogeneic TRIM5α colored yellow, and co-migrating TRIM5α proteins colored green. **(C)**. Graph of relative virus release (virus in supernatant/virus in cells and supernatant at 6 h) is presented. **(D)**. A graph of relative virus-specific infectivity (virus infectivity after a 6-h collection period normalized by CA signal). Graphs shown in **(C)** and **(D)** represent averages of three independent experiments, and error bars indicate standard deviation.

The strength of AgmTRIM5α restriction of HIV-1 and SIV in both the cell-free and, especially, the cell-to-cell replication assays is unexpected since its influence on single-round infection was fairly modest. One formal possibility for the discrepancy between the replication and single-round infection results is that the expression of xenogeneic TRIM5α in our cells decreases virus production. Previous reports indicated that xenogeneic expression of TRIM5α can reduce the amount of infectious virions produced from cells [51,62], though these observations remain controversial [20,52,63]. To formally rule out lower virion production from infected cells by our xenogeneic TRIM5α expression approach as a possible mechanism for our results, we investigated whether the expression of AgmTRIM5α or gorTRIM5α reduces virus release from infected cells. We transduced two human CD4^+^ T-cell lines, Clone 4 [64] (cloned from HIV-infected H9 cells) and E11S [65] (cloned from SIV-infected HuT 78 cells), which produce high constitutive levels of HIV-1 or SIV, respectively with the SMS-hAgmT or SMS-hgorT vectors. Transductants were selected for puromycin resistance and confirmed by GFP fluorescence, producing the Clone 4 lines Cl4/hAgmT and Cl4/hgorT and the E11S lines, E11S/hAgmT and E11S/hgorT. Quantitative NIr-immunoblot analysis of Clone 4 and E11S transductant cell lysates revealed that exogenous TRIM5α was expressed at least 2- to 5-fold more than the endogenous human protein (Figure 5B), higher than the 1- to 2-fold level observed in the JR5 transductants (Figure 1B).

The production of virions from Clone 4 and E11S and their xenogeneic TRIM5α transductants was measured by isolating virions and cells after a 6-h incubation and analyzing sample lysates by quantitative NIr immunoblotting with CA antibodies. From these data, the release rate was calculated as the amount of CA fluorescent signal in the supernatant divided by the total signal detected, i.e. that amount present in both the virions and cells. The blots showed little difference in the production of virions from untransduced and transduced cell lines (Figure 5B). Comparing calculations from three independent experiments showed no significant differences in release between the untransduced and TRIM5α-expressing cell lines. Taken together, these data indicate that virion production is not reduced by xenogeneic TRIM5α transduction and thus is not a factor in the increased potency of AgmTRIM5α restriction of replication.

### Xenogeneic TRIM5α expression has no effect on infectious virion production

Even though release is normal, it is possible that the virus produced from our xenogeneic TRIM5α transductants is less infectious than the virus produced from the untransduced cells because of a subtle defect in assembly or maturation. To examine this possibility, the specific infectivity of the virions produced by the Clone 4 and E11S cell lines was measured by the Tat-complementation TZMbl beta-galactosidase focus-forming assay and normalized by the amount of CA in the medium as measured by NIr immunoblot quantitation to calculate specific infectivity of each sample. The results showed that the expression of xenogeneic TRIM5α had no measurable effect on the specific infectivity of the virions produced from cells (Figure 5C). This finding, together with the release data, formally rule out late replication defects as the reason for the potency of AgmTRIM5α replication restriction.

## Discussion

Our results identify TRIM5α from *tantalus* African green monkey as an effective restriction factor of HIV-1 and SIV replication in CD4^+^ T cells, producing very potent restriction of both HIV-1 and SIV replication, approximately ≥10^-3^ in the cell-free challenge and ≥10^-2^ in the more rigorous infected cell challenge system. The ability of AgmTRIM5α to restrict in the context of a cell-to-cell challenge is remarkable considering that this mode of infection is up to 1000-fold more efficient than the cell-free infection challenge [48,55-61]. In comparison, rhTRIM5α fails to restrict HIV-1 replication in our cell-to-cell challenge assay, even though it exhibits essentially equivalent levels of restriction in our short term infection assay as AgmTRIM5α. Furthermore, as discussed above, primary human CD4^+^ T cells transduced with rh*TRIM5α* or stabilized human *TRIM5α* genes restrict HIV-1 replication, yet fail to do so in cell-to-cell contact with untransduced infected cells [10,48], conditions similar to our cell-to-cell challenge. Similarly, Ohkura et al. found that N-tropic murine leukemia virus produced restriction resistant mutants in target cells expressing either human or rhesus TRIM5α only when co-cultured with unmodifed cells, indicating a low level of infection in the mixed cultures that was absent with the modified cells alone [66,67]. Therefore, AgmTRIM5α is unique among TRIM5α proteins studied to date in that it can restrict both HIV-1 and SIV in the severe test afforded by the cell-to-cell challenge in the presence infected non-expressing cells.

It is important to note that after the initial challenge, our cell-free experiments develop a cell-to-cell infection component. After the inoculum is absorbed, the first generation of productively infected cells can initiate the next round of replication by both cell-free and cell-to-cell infection. Because all of the cells express AgmTRIM5α in the cell-free assay, the initial levels of infected cells are low, thus limiting the opportunity for cell-to-cell transmission. It is important to consider that TRIM5α-mediated restriction is saturable, resistance being overcome by the presence of enough capsid cores to bind and sequester the xenogeneic TRIM5α and neutralize restriction. When every target cell in a culture expresses AgmTRIM5α enough virus needs to enter the cells to saturate the xenogeneic TRIM5α. Since the amount of virions produced from the initial infection is low in the presence of AgmTRIM5α, the resulting MOI, either cell-free or cell-to-cell, generated by the cells infected in the second round is insufficient to saturate TRIM5α in many cells and fails to support spread of infected cells in the subsequent rounds of replication. In contrast, our cell-to-cell experiments initially have infected JR5G cells, thus provide a stronger challenge to restriction which more readily overwhelms the resistance in AgmTRIM5α-expressing targets, resulting in a lower, yet still considerable, level of restriction.

The nature of TRIM5α restriction also can explain the counter-intuitive relationship between the single-round infection and replication assays. In most cases of retroviral inhibition by other resistance factors or anti-viral compounds, the decrease in infectivity follows linear kinetics: the level of inhibition is directly proportional to that of the agent/defect. In contrast, the suppression of TRIM5α restriction exhibits multi-hit kinetics: many intact capsid cores need to enter the cell to saturate the cooperatively binding TRIM5α, before the strong anti-viral block to infection is removed. Thus, capsid core–mediated saturation of TRIM5α restriction follows logarithmic kinetics with a strong, nearly absolute, block at lower MOIs that initially has small marginal reductions in restriction with increasing virus exposure. Because restriction requires cooperative TRIM5α binding to intact capsid cores [41,42,68], as the amount of restricting TRIM5α in the cell is reduced by binding to the cores, incremental dosages of capsid cores have larger marginal effects on saturating TRIM5α restriction [41,68-71]. After TRIM5α is saturated, infection occurs without restriction. Therefore, subsequent rounds of infection initially face a near-absolute block mediated by TRIM5α restriction which requires enough capsid cores to overcome this restriction hurdle and productively infect cells. In this way, a modest block has a cumulative effect, being amplified by the amount of virus needed to surmount the restriction hurdle at each round of replication. Indeed, Ohkura et al. observed nearly absolute restriction to replication in an N-tropic murine leukemia virus/rhTRIM5α system that was negligible at a 10-fold higher MOI, consistent with this restriction hurdle model [66]. In addition to the inherent nature of TRIM5α restriction, other cellular and viral factors can come into play. For instance, a spreading infection can also be limited by virus-induced cell death and the spontaneous decay of virions in the cell culture supernatant [72,73], both of which remove sources of infection.

Given this cumulative restriction hurdle model, the failure of gorTRIM5α to effectively restrict SIV replication in either assay can be explained by the lower efficacy of gorTRIM5α restriction observed in the single-round and short term infection assays. For gorTRIM5α, the restrictive barrier to infection, although reducing SIV single-round infection by 70%, does not appear to present a high enough of a restriction hurdle to have a cumulative effect for replication.

Taken together, AgmTRIM5α appears to be superior to other TRIM5α in restricting both HIV-1 and SIV when expressed at near-physiological levels. Despite observing inhibition of infection that was comparable to that of AgmTRIM5α in a single-round infection assay, rhTRIM5α failed to restrict in our cell-to-cell assay. While these two proteins are fairly homologous, AgmTRIM5α contains a 37–amino acid region with a unique 20-amino acid duplication in the B30.2(SPRY) domain which forms the basis of SIV_mac_ restriction [22,30] and is likely to be a factor for its superior potency against HIV-1 replication. Additionally, the basic restriction mechanism for AgmTRIM5α restriction appears to have different requirements than those of rhTRIM5α, notably E3 ubiquitin ligase activity [32]. With its B30.2(SPRY) duplication, AgmTRIM5α is unique among primate TRIM5α proteins [24], and this feature may be the reason for the exceptional restriction of cell-to-cell infection. Fine mapping of the critical residues in this region of B30.2(SPRY) responsible for the elite restriction by AgmTRIM5α and their interactions with HIV-1 capsid should yield important information about the mechanism for effective TRIM5α restriction.

The restrictive properties of TRIM5α have been proposed as the basis of anti-viral therapies [1,2,8,10,49]. Our results showing effective AgmTRIM5α-mediated restriction of HIV-1 and SIV_mac239_ in CD4^+^ T cells are consistent with the use of the TRIM5α restriction mechanism as an anti-HIV/SIV approach. Indeed, Shi et al. have described a small-molecule inhibitor that prematurely uncoats the HIV-1 capsid core after viral entry [74]. While TRIM5α restriction is clearly saturable and thus not an absolute block to infection, it can and does slow the spread of virus in target cells. Indeed, experiments in primary rhesus CD4^+^ T cells show that AgmTRIM5α offers significant protection from SIV_mac239_ thereby enhancing their antiviral function (SJ, MTT, VIA, CO, and DEO manuscript submitted). Our results open up the SIV/rhesus macaque model for in vivo studies of TRIM5α restriction. Of course, one consideration for using any xenogeneic TRIM5α as a protective therapy is whether it will induce an adverse immune response in the recipient [8]. While this is likely to preclude the direct use of AgmTRIM5α as a therapeutic, the nature of TRIM5α is a facet of AIDS virus biology that is important to understand and potentially harness.

## Conclusions

Using a dilution end-point replication assay, we find that AgmTRIM5α exhibits a greater than 10^3^-fold level of restriction of HIV-1 and SIV_mac239_ replication in a transformed human CD4^+^ T-cell line when expressed at near physiological levels. This level of restriction was markedly greater than the restriction observed in single-round or short term infectivity assays, indicating restriction building up cumulatively over many rounds of replication. Additionally, AgmTRIM5α could restrict HIV-1 and SIV_mac239_ replication in co-culture with infected normal cells. In contrast, rhTRIM5α failed to effectively restrict HIV-1 in co-culture, a finding previously observed for both rhTRIM5α and a stabilized mutant human TRIM5α exhibiting an extended half-life. Thus, AgmTRIM5α is unique among the TRIM5α proteins tested to date. These results provide a basis for directly examining TRIM5α restriction in the SIV/rhesus macaque model to better understand and potentially utilize this anti-AIDS virus mechanism in the clinic.

## Methods

### Viruses and cells

293T human embryonic kidney, Phoenix RD114 (clone 22) [75] and TZM-bl [76] (also known as JC53-BL clone 13) cells were cultured in Dulbecco’s modified Eagle’s medium supplemented with 2 mM L-glutamine, 100 U per ml penicillin, 100 μg per ml streptomycin and 10% vol/vol fetal bovine serum. Jurkat CCR5, Clone 4, and E11S cell lines were cultured in RPMI 1640 with the same supplements as those in the 293 T cell medium. All cell culture products were obtained from Life Technologies, Inc. All transient transfections of 293T cells were carried out using TransIt293 (Mirus Bio Corp.). HIV-1, SIV_mac239_, and SIV_Agm_ stocks were produced by transfecting HEK293T cells with pNL4-3 [77], p239_mac_SPXL (gift of Ronald Desrosiers) [78-80], or pSIV_AgmTan-1_ [81] (obtained through the National Institutes of Health [NIH] AIDS Reagent Program, Division of AIDS, National Institute of Allergy and Infectious Diseases [NIAID], NIH, from Drs. Marcelo Soares and Beatrice Hahn). Viral and viral vector supernatants were passed through a 0.45 μ filter before use. Short term infections were carried out in the presence of 2 μg/mg hexadimethrine bromide at an MOI of 0.05.

### Retroviral vectors and generation of cell lines

Agm- and gor*TRIM5α* genes [22] were a kind gift of Theodora Hatziioannou (Aaron Diamond AIDS Research Center, New York, NY). The rhesus (rh) gene (Cat# 10072) was obtained from the NIH AIDS Repository Program, Pathogenesis and Basic Research Branch, Division of AIDS, NIAID, Bethesda, MD). Agm-, gor-, and rh*TRIM5α* genes were cloned into a modified MSCV PIG retroviral vector (obtained from Addgene , plasmid 18751, deposited by Scott Lowe) with an N-terminal hemagglutinin epitope tag (YPYDVPDYA) added to the *TRIM5α* coding sequence to produce pSMS-Agm, pSMS-gor, and pSMS-rh vector constructs that also express both the puromycin resistance and GFP genes. The three TRIM5α genes were also cloned into the pBabe-puro retroviral vector [82], obtained from Addgene, plasmid 1764, contributed by Bob Weinberg to produce pBabe-Agm, pBabe-gor, and pBabe-rh vector constructs. Retroviral vectors were packaged by transient transfection of Phoenix RD114 packaging cells [75] (kind gift of Hans-Peter Kleim, Fred Hutchinson Cancer Research Center, Seattle, WA). GFP-expressing JR5 cells were produced by transduction with a GAE-SFFV-GFP-WPRE lentiviral vector DNA (kind gift of Francois-Loic Cosset, Inserm Lyon, France) [83] that was modified by fusion of a P2A-puroTK cassette to the C-terminus of GPF to produce the GSPK vector that expresses GFP, and a puromycin-herpes thymidine kinase fusion gene. The GSPK lentiviral vector was produced by transiently cotransfecting HEK293T cells with the pGSPK DNA construct, and pAD-SIV4 packaging plasmid [83] (kind gift of F-L Cosset) and pLPVSVG, at a ratio of 4.5:4.1:1 by mass. GFP-expressing LKO HIV lentiviral vector (GE Healthcare Dharmacon, Piscataway, NJ) was packaged using the Virapower packaging system (Life Technologies) in transfected 293 T cells. Supernatants were collected 48 h post-transfection and passed through a 0.45 μ filter. Transductions were carried out in the presence of 2 μg/mg hexadimethrine bromide and Viromag R/L reagent (Boca Scientific, Inc.), the latter reagent concentrating supernatants to deliver a higher MOI. TRIM5α vector transductants were selected for puromycin resistance. Puromycin (Sigma-Adlrich Inc.) selection was carried out at 2 μg/ml. For single-round infectivity assays, transductions were carried out at an MOI of 0.05.

### Virus and cell lysate preparation and isolation

HIV-1-containing cell culture supernatants were clarified by centrifugation at 2,000 × *g* for 5 min at 4°C, and then samples directly lysed in a one-half volume of SDS-PAGE loading buffer (250 mM Tris-Cl pH 6.8, 8% sodium dodecyl sulfate, 4% vol/vol β-mercaptoethanol, 40% vol/vol glycerol, and 0.02% bromophenol brilliant blue). SIV-containing cell culture supernatants were clarified and then concentrated 10-fold using the Lenti-X virion concentration reagent (Clontech Laboratories) according to the manufacturer’s instructions. Precipitated virions were lysed directly in SDS-PAGE gel loading buffer. Cell samples were collected and washed once with ice-cold Dulbecco’s phosphate-buffered saline solution without Ca^+2^ or Mg^+2^ (D-PBS, Life Technologies) and then lysed with prechilled 10 mM Tris HCl pH 7.5, 0.1% SDS, 5 μg of phenylmethanesulfonyl floride and 4 U/μl of Omnicleave® nuclease (Epicentre Biotechnologies), incubated for 2 h at 4°C and treated with an equal volume of SDS-PAGE gel loading buffer.

### Near infrared (NIr) immunoblots

Samples were separated by SDS-PAGE electrophoresis and blotted onto PVDF-FL membranes (Millipore) using a semi-dry apparatus as previously described [84]. Blots were blocked for at least 1 h in Odyssey blocking buffer (LI-COR Biosciences) and then incubated in blocking buffer with one or two primary antiserum (a)/antibody(ies) for at least 2 h, typically overnight. Blots were washed twice with blocking buffer for 10 m then incubated with the appropriate donkey IRDye 800CW and/or IRDye680 LT fluorescently labeled secondary antibodies (LI-COR) at a 1:10,000 ratio vol/vol in blocking buffer for at least 2 h. Blots were washed five times for 10 min each time in blocking buffer, and then analyzed with an Odyssey infrared imaging system (LI-COR) using a laser intensity of between 1 and 5. Signal densities of bands were measured by the Odyssey 3.0 application software. All blotting steps were carried out at room temperature. Monoclonal antibodies used were anti-rhTRIM5α, clone 3F1-1-9, (Cat# 12271, NIH AIDS Reagent Program) and anti-beta actin (Cat# 60008-1-Ig, ProteinTech Group). Primary goat antiserum against HIV p24^CA^/SIV p27^CA^ (goat #81) was obtained from the AIDS and Cancer Virus Program, Frederick National Laboratory for Cancer Research , Frederick, MD.

### Virus release assays

Virus release was carried out as previously described [85]. Briefly, 5 × 10^6^ cells were collected by centrifugation, washed twice with PBS and then placed in 5 ml of pre-warmed medium and incubated at 37°C. At 6 h, virus and cell lysates were prepared and analyzed by quantitative CA near infrared NIr immunoblot. Release factors for the viruses were determined by dividing the measured CA fluorescence values for the virus samples (arbitrary units) by the total Gag signal (virus + cellular Gag) to produce a release factor. The cellular Gag values were corrected for loading and cell extract processing differences by normalizing signals from actin staining with a second color.

### Specific infectivity assays

Specific infectivity assays were carried out over a 6-h time period using the same procedure as the virus release assay presented above with clarified supernatants assayed for infectivity and virion lysate samples were prepared. Infectivity assays were performed using the TZM-bl single-round *lacZ* Tat complementation assay as previously described [86]. CA present in the supernatant was measured by CA NIr immunoblot. Specific infectivity was calculated dividing the virus titer by the CA band signal, expressed in blue cell-forming units per arbitrary CA fluorescence value. Heat inactivation was carried out at 70°C for 20 min.

### Flow cytometry analysis of infected cell lines

For each sample, medium containing 1-2 × 10^6^ cells was centrifuged at 400 × *g* for 5 min and fixed in 0.5 ml of 4% wt/vol paraformaldehyde in D-PBS (Life Technologies) for 20 min at room temperature followed by the addition of 3.5 ml permeabilization solution (PS), 0.1% (wt/vol) saponin (Sigma-Aldrich) in D-PBS, and incubation at room temperature for 10 min. Cells were then collected by centrifugation at 550 × *g* for 5 min, followed by 2 washes with PS. Cells were then stained with KC57 antibody for HIV-1 CA (Beckman-Coulter, Inc.) or 2 F12 antibody for SIV CA (Quality Biological, Inc., Gaithersburg, MD) in 100 μl of PS for 30 min in the dark at 4°C, washed twice in 4 ml of PS and then resuspended in 200 μl of PS, and analyzed immediately by an LSRII flow cytometer (BD Biosciences) Data analysis was performed using FCS Express software (De Novo Software).

### Statistical analysis

Single-round and short term infectivity data where analyzed using the Student’s t-Test function in Excel (Microsoft Inc.) with paired two-tailed parameters.

### Ethical approval

All research was carried out under approval by the NCI at Frederick Institutional Biosafety Committee # 11-03 superseded by #14-23.
